# Back pain outcomes in primary care following a practice improvement intervention:- a prospective cohort study

**DOI:** 10.1186/1471-2474-12-28

**Published:** 2011-01-27

**Authors:** Alan C Breen, Eloise Carr, Jennifer E Langworthy, Clive Osmond, Louise Worswick

**Affiliations:** 1 Institute for Musculoskeletal Research and Clinical Implementation, Anglo-European College of Chiropractic, Parkwood Road, Bournemouth, BH5 2DF, UK; 2 School of Health and Social Care, Bournemouth University, Christchurch Road, Bournemouth, UK; 3 MRC Epidemiology Resource Centre, University of Southampton, Southampton General Hospital, Southampton, UK

## Abstract

**Background:**

Back pain is one of the UK's costliest and least understood health problems, whose prevalence still seems to be increasing. Educational interventions for general practitioners on back pain appear to have had little impact on practice, but these did not include quality improvement learning, involve patients in the learning, record costs or document practice activities as well as patient outcomes.

**Methods:**

We assessed the outcome of providing information about quality improvement techniques and evidence-based practice for back pain using the Clinical Value Compass. This included clinical outcomes (Roland and Morris Disability Questionnaire), functional outcomes, costs of care and patient satisfaction. We provided workshops which used an action learning approach and collected before and after data on routine practice activity from practice electronic databases. In parallel, we studied outcomes in a separate cohort of patients with acute and sub-acute non-specific back pain recruited from the same practices over the same time period. Patient data were analysed as a prospective, split-cohort study with assessments at baseline and eight weeks following the first consultation.

**Results:**

Data for 1014 patients were recorded in the practice database study, and 101 patients in the prospective cohort study. We found that practice activities, costs and patient outcomes changed little after the intervention. However, the intervention was associated with a small, but statistically significant reduction in disability in female patients. Additionally, baseline disability, downheartedness, self-rated health and leg pain had small but statistically significant effects (p < 0.05) on follow-up disability scores in some subgroups.

**Conclusions:**

GP education for back pain that both includes health improvement methodologies and involves patients may yield additional benefits for some patients without large changes in patterns of practice activity. The effects in this study were small and limited and the reasons for them remain obscure. However, such is the impact of back pain and its frequency of consultation in general practice that this kind of improvement methodology deserves further consideration.

**Trial registration number:**

ISRCTN: ISRCTN30420389

## Background

Back pain is one of the UK's costliest and least understood health problems, whose prevalence still seems to be increasing [1]. General practice is the most common destination of those who consult a practitioner and back pain has the highest individual consultation prevalence of all the musculoskeletal disorders [2,3]. However, multiple studies have identified problems with the adoption of the evidence as presented in back pain guidelines [4-7].

Qualitative studies into general practitioner (GP) attitudes to back pain have suggested that including education and feedback could be promising [8-11], However, randomised trials have been less positive. Three such trials found that GP education did not by itself improve patient outcomes in terms of symptoms, disability, or satisfaction with care [12] although it did modestly increase guideline consistent behaviour [13], and GP confidence [14]. Engers et al also conducted a cluster randomized trial of a GP educational intervention to promote adoption of the Dutch back pain guidelines [15]. This study found little change in management patterns. However, an Italian cohort study which assessed the efficiency of an educational program on the clinical behaviour of doctors for the diagnosis of low back pain reported a shift of priorities from diagnosis to communication with patients [16].

Two occupational studies of physician education showed slightly more positive results. A Norwegian randomised controlled trial of the implementation of 'Active Sick Leave' (ASL - a scheme to facilitate the use of return to work with modified activities), used educational workshops and literature to help promote general practitioner adoption for patients who were off work with back pain [17]. This trial found that a significantly higher proportion of people used ASL when they and their doctors were telephoned and reminded, but the effects were attributed more to contact with patients than with doctors. In a further US occupational study by Derebery et al evaluated the effect of an educational intervention on physician management of employees with back pain and found that it did reduce the percentage of restricted work and lost-time cases for some patient categories [18]

So far there have been no UK studies of GP educational interventions to improve the management of back pain, although one randomized trial of a posted information package reported a positive shift in beliefs and behaviours in chiropractors, osteopaths and musculoskeletal physiotherapists [19].

Researching care improvement benefits from the documentation of current care and prognostic factors, as well as the effects of interventions. One systematic review of randomised trials that examined both of these for back pain found inadequate data to describe the care given and a lack of studies that assessed the role of prognostic factors [20]. It was also recognised that 'usual care' was often highly variable. Post-hoc analysis of one large back pain trial that included GP care found that although duration of pain did not affect the clinical outcome, age, work status, age of leaving school, pain, disability, quality of life, and patient beliefs all did [21]. Assessment of guideline implementation strategies should therefore take these factors into account.

One ethnographic study in the UK found that in the presence of organisational demands and constraints, iterative negotiations and informal networking may be a promising approach [22] and a methodology for doing this using an improvement model that combines practice-based learning, inter-professional networking and continuous quality improvement cycles has been suggested [23].

In order to test the effects of such a combined inter-professional approach, this study used facilitated 'action-learning' to promote evidence-based back pain management, along with practical knowledge of health care quality improvement [24]. Action-learning encourages reflection on actions and learning from each other. Used with a group of different professionals it can address organisational change [25] and has been used in a range of settings including mental health [26], managers on leadership programmes [27] and care homes [28].

In the present study, 9 practice teams, each with a patient, attended a series of 8 half-day workshops over 9 months. For our outcomes evaluation we used the Clinical Value Compass whose components are: clinical outcomes, functional health status, satisfaction against need and total costs [29]. This tool was developed to track and evaluate measures which could help teams improve performance of care delivery by linking the processes of care with patient outcomes. It has been used in a range of clinical settings and countries including Canada [30], Australia [31] and the United States [32]. In an ambitious improvement project, Deyo et al, working with healthcare organisations delivering back pain management, also identified a range of outcome measures, but limited detail was provided in terms of outcome data and these were not framed within the Clinical Value Compass[33].

We also recorded GP interventions, referrals and their costs in a parallel study of the same practices over the same time period. The purpose of this study was twofold:

1. to assess changes in patient outcomes following a practice improvement intervention that included patients in the improvement learning, taking account of prognostic factors and 2. to identify any changes in practice care patterns for back pain after such an intervention.

## Methods

### Population

#### Patient recruitment

Nine practices from two Primary Care Trust localities in the South of England recruited two cohorts of patients who attended for back pain. The first cohort was recruited in the 12 months before practice teams had attended 8 half-day workshops over nine months starting in March 2008. The second cohort was recruited over the 15 months following the workshops. Patients were recruited by the general practitioners, who explained the study, obtained consent to pass their contact details to a co-ordinator and gave consenting patients an information sheet.

#### Inclusion and exclusion criteria

Inclusion criteria were: age 18-65, low back pain, with or without leg pain, back pain of less then 12 weeks duration and no previous spinal surgery. Patients were not eligible if they had serious spinal pathology, traumatic onset of their pain, were pregnant, had severe depression, had constant pain lasting more than 6 months or had compensation or litigation proceedings pending.

#### Characteristics of the practice teams

Each team had up to 6 members, each comprising up to three doctors, plus various combinations of people involved with the practice, including physiotherapists, receptionists, practice nurses, practice managers and patients. All practice teams included at least one patient. A bursary was provided for each practice team to offset costs such as provision of locum cover.

### Practice database study

Concurrent with the collection of questionnaires from patients, a second study was conducted of practice activity for back pain patients as recorded in practice computer databases. For this, the computer databases (EMIS, Vision, ISOFT and Synergy) of all practices were extended to include a range of new variables to be recorded if the Read Code N142 'low back pain' was entered. Doctors then logged their consultation activities for all back pain patients at each visit, including sickness certification, referrals and their own interventions. Prescribing of oral medications was not included. All 9 practices, populated by 40 doctors, participated in this data collection process. Costs were attributed to each chargeable activity from national and local NHS tariffs for 2008-9. This constituted the fourth Clinical Value Compass component. Medication costs were not included.

### Intervention

During the workshops, both the principles of quality improvement and the latest evidence for back pain management, based on the European Acute Back Pain Guidelines [34], were introduced. This was based on information provided through fast feedback questionnaires from the practice teams at the end of the previous workshop in order to ensure that ownership of the learning remained with them. The forms identified speciality areas and expertise was brought in from external sources. These included speakers who covered communication skills, psychological aspects of pain, pain management, examination skills, Expert Patient Programme, back pain support groups and bio-psycho-social aspects of back pain (see Additional file 1 for the content of workshops)..

Each practice team also identified its own individual priorities for improving care and generated at least one improvement project of its own, using a plan, do, study, act (PDSA) methodology. Practices were also supported by a project Wiki. This was a private section of the LIMBIC website (http://www.limbic.org.uk) which posted information and commentaries by the project team and other participants. It was central to communication about specific project activity; improvement projects and the sharing of ideas. A Quality Improvement Facilitator also visited practices to help with problems and answer queries. This Facilitator played a key role in supporting practices with the development of their improvement projects, the sharing of ideas with other practices, and the use of the Wiki to provide access to improvement tools.

During the phase of data collection from patients, the co-ordinator made telephone contact with them within one week, obtained consent and the baseline questionnaire was then completed. Eight weeks later, a follow-up questionnaire was completed. The study received ethical approval from the (Somerset NHS Ethics Committee Reference 07/H0205/36), and was registered on the Current Controlled Trials database (ISRCTN30420389).

### Outcome Measures

**Figure 1:**
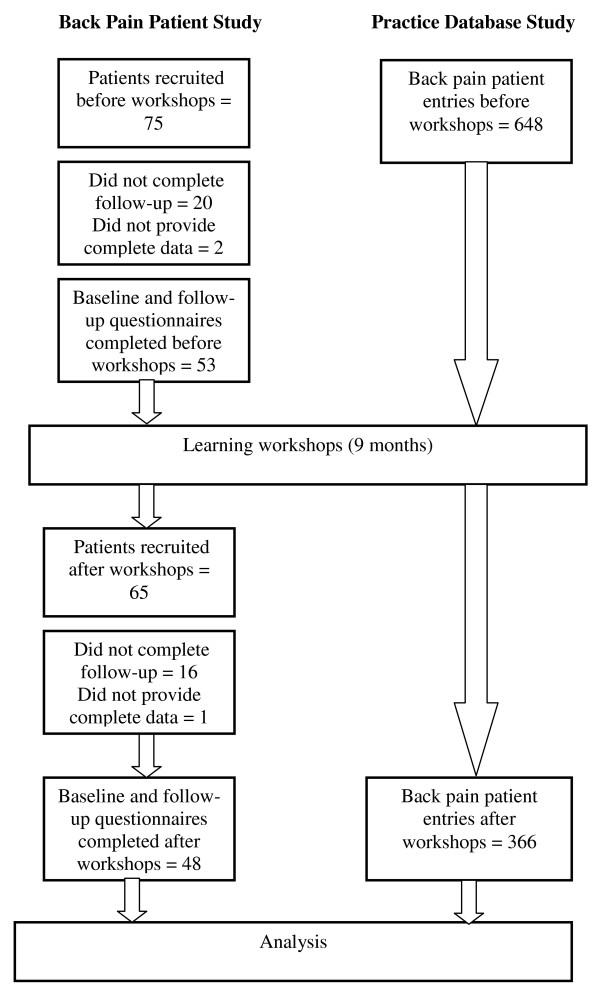
**Recruitment and Data Collection**.

In the back pain patient study, patient outcomes were assessed with the cohort split into before and after workshop groups (Figure 1). The patient baseline questionnaire contained demographic information, prognostic variables and the Roland and Morris Disability Questionnaire (RMDQ) [35]. This was the primary outcome measure for the clinical outcome in the Value Compass [29]. Secondary outcomes comprised a 10-point numerical rating scale for pain, Deyo's symptom frequency and bothersomeness index [36] and the general health, mental health, vitality and social function subscales of the SF-12 [37]. These made up the functional status measures of the Value Compass. Eight weeks later, participating patients received a follow-up questionnaire with the same measures, plus a numerical rating global improvement/deterioration scale, the Patient Satisfaction Scale [38] and a single satisfaction question [39] which constituted the third measure of the Value Compass. All data were entered into an SPSS (V17.0) file.

### Analysis - back pain patient study

Demographic and prognostic variables for each Cohort were analysed descriptively. Significance of differences of change scores for interval or categorical data between cohorts was determined using 2-sided Mann-Whitney and Fisher Exact tests. Significance for all tests was set at the 5% level.

We combined data from both cohorts and used multiple linear regression analysis to model the RMDQ score at follow-up, always including as predictors age, sex, the baseline RMDQ score and a dummy variable that identified whether the patient was seen before or after the series of workshops. The regression coefficient for the last term measured the effectiveness of the workshop. Other variables that might influence the follow-up RMDQ score were introduced in a forwards stepwise manner - the most significant variable from the remaining set being introduced at each stage until the introduction of a new variable was not statistically significant at the 5% level.

Differences in the proportions of patients improved were also calculated. These were determined using ROC curve analyses to determine the RMDQ cut-off scores for an improvement of at least 2 out of 10 on the global improvement question from the follow-up questionnaire and, alternatively, as the same reduction of severity on the visual analogue pain scales between baseline and follow-up [40].

Ten questions from the Patient Satisfaction Scale were grouped under the subsets of; Information, Caring and Effectiveness [38]. An additional overall satisfaction question was included and the median scores in patients seen before and after workshops were analysed for differences using the Mann-Whitney test.

### Analysis - practice database study

Data were transferred from practice databases into Microsoft Excel (2003 Version) and SPSS (V17.0) files for analysis. Items were tallied by practice for the pre and post intervention cohorts and analysed by cohort. The rates of the various practice activities per patient were calculated by dividing the number of times the activity was used by the number of patients in the cohort. The average cost of each activity per patient was calculated by multiplying its relevant NHS tariff for 2008-9 by the rate for that activity. The average total cost of an episode was estimated by summing these costs. Differences in the proportions of patients who received the most frequent care activities were compared using the Fisher Exact test.

## Results

### Back pain patient study

Seventy-five patients were recruited for the pre-intervention phase (Cohort 1) and 65 to the post-intervention phase (Cohort 2) (Figure 1). Twenty Cohort 1 and 16 Cohort 2 patients dropped out and 3 and 1 respectively did not provide complete data. Data were analysed for 53 patients in Cohort 1 and 48 in Cohort 2 (Table 1). The mean age (sd) of the combined cohorts was 47 (10.991) and 52% were female. The mean duration of their pain was 3.69 weeks (2.922), however in only 24% was this the first episode. Mean pain severity was 7.25/10 (1.172) and disability 11.46/24 (5.063).

**Table 1:** Baseline Demographics and Prognostic Indicators

	Mean(SD)		
**Variable**	**Cohort 1 (n = 53)** **(pre-workshops)**	**Cohort 2 (n = 48)** **(post-workshops)**	**Significance (p)**

Age	47.02 (10.589) *(range 22-69)*	47.48 (11.526) *(range 18-64)*	0.693*
Gender (% F)	51%	52%	0.909**
Age left school			
• <16 yrs	34% (n = 18)	35% (n = 17)	0.880**
• ≥16 yrs	66% (n = 35)	65% (n = 31)	
Duration (weeks)	3.47 (2.791)	3.94 (3.069)	0.523**
Chronicity (proportion of previous 12 m)			
• <50%	57%	52%	0.655**
• ≥50%	13%	31%	0.032**
• First episode	30%	17%	0.118**
Severity (/10)	7.38 (1.632)	7.10 (1.882)	0.538*
Disability (/24)	10.91 (5.289)	12.06 (4.782)	0.195*
Job satisfaction (enjoy job)	81%	60%	0.014**
Bothersomeness			
• LBP (/5)	3.83 (0.955)	3.79 (1.031)	0.896*
• Leg pain (/5)	2.47 (1.475)	2.62 (1.071)	0.588*
• Moderate-severe leg pain (3-5/5)	42% (n = 22)	52% (n = 25)	0.296**
Interference with work (1-4)	2.98 (0.990)	2.96 (1.071)	0.908*
Life impact (0-4)	0.36 (0.834)	0.52 (0.031)	0.415*
Cut down activities (/28 days)	8.13 (6.964)	11.29 (8.417)	0.060*
Cut down work (/28 days)	5.29 (6.141)	6.41 (7.305)	0.666*
Downheartedness (/5)	1.51 (1.250)	1.98 (1.407)	0.067*

* = Mann-Whitney, ** = Fisher Exact test

Both Cohorts were representative of an adult acute and subacute non-specific back pain population, although Cohort 2 had significantly more people who reported pain lasting at least 50% of the days of the previous 12 months (Fisher Exact test, 2-sided p = 0.032) and significantly fewer who said they enjoyed their jobs (Fisher Exact test, 2-sided p = 0.032). (However, for the latter, many people were not in paid employment.) There were no other significant differences between Cohorts.

### Regression analysis - primary outcome

Multiple regression analysis of the pooled data revealed that baseline (RMDQ) score, downheartedness, self-rated health and leg pain had small but statistically significant effects on follow-up RMDQ scores in various groups (Table 2). When these were controlled for in the regression, people who attended for back pain in the post-intervention period had follow-up RMDQ scores that were 1.43 (95% confidence interval -0.38-3.25) lower than those in the pre-intervention period. The corresponding figures for males and females were -1.14 (1.89 to -4.17) and -2.90 (-0.47 to -5.32) respectively, giving a statistically significant effect for females. Higher baseline disability had a small, but statistically significant effect on follow-up disability in males.

**Table 2:** Multiple regression analysis: prognostic factor effects on follow-up RMDQ scores

Prognostic factor†	Both sexes (n = 101)		Males (n- = 49)		Females (n = 52)	
	**Effect on RMDQ (95%CI)**	**(p)**	**Effect on RMDQ (95%CI)**	**(p)**	**Effect on RMDQ (95%CI)**	**(p)**

**In post-workshops Cohort (y/n)**	-1.433 (-3.25-0.39)	0.121	-1.142 (-4.17-1.89)	0.451	-2.897 (-5.32- -0.47)	0.020
**RMDQ baseline score (/24)**	0.299 (0.10-0.50)	0.003	0.362 (0.05-0.68)	0.026	0.086 (-1.9-0.36)	0.535
**Downhearted (/5)**	0.991 (0.26-1.72)	0.008	0.503 (-0.67-1.68)	0.451	1.634 (0.66-2.61)	0.002
**Self-rated health (/4)**	-1.245 (-2.27- -0.22)	0.018	-1.368 (-2.85-0.11)	0.069	-1.524 (-3.09-0.05)	0.056
**Leg pain bothersomeness (/4)**	0.644 (-0.03-1.32)	0.063	0.323 (-0.68-1.33)	0.520	1.431 (0.43-2.44)	0.006
**Episode duration (/12)**	0.270 (-0.06-0.60)	0.106	0.404 (-0.14-0.95)	0.140	0.074 (-0.52-0.37)	0.741

† For every higher category of each prognostic factor, the 'Effect' in RMDQ units, rises by the amount shown. For example, the figure 0.991 for 'Downhearted' under 'Both sexes' means that the follow-up RMDQ score is 0.991 units higher for every higher category of Downheartedness.

### Proportion of patients improved

A higher proportion of patients who attended the practices after the workshops improved (0.64 vs 0.51) in terms of the global scale, but not the pain scale (0.69 vs 0.70). Neither was statistically significant, although such small changes observed at a population level could be very important to individual patients.

### Functional outcomes

Change scores for functional outcomes are shown in Table 3. There were small differences in improvement in global outcome and activity after the workshops which were not statistically significant.

**Table 3:** Functional outcomes

Outcome (possible change)	Median change		Significance
	Cohort 1	Cohort 2	(2-sided p)
Pain severity (/9)	-4.0	-4.0	0.864
Symptom frequency scale (Deyo 1988)			
Back pain bothersomeness	-1.0	-1.5	0.625
Interference with work	-2.0	-1.5	0.675
Life impact (/4)	-1.0	-1.0	0.364
Cut down activity (/28	-2.0	-5.0	0.252
Cut down work (/28)	-1.0	-1.0	0.607
SF-12 subscales			
General health (/4)	0.0	0.0	0.180
Interference with normal work (/4)	-1.0	-1.0	0.298
Feeling calm (/4)	0.0	0.0	0.891
Having energy (/4)	0.0	-1.0	0.190
Feeling downhearted (/4)	0.0	-1.0	0.151

### Satisfaction

There was no statistically significant difference in post-treatment satisfaction scores between patients who consulted before and after the workshops (Table 4).

**Table 4:** Satisfaction with general practitioner care

Outcome (possible change)	Median score		Significance
	**Cohort 1**	**Cohort 2**	**(2-sided p)**

Information giving (/4)	2.3	2.3	0.393
Caring (/4)	2.0	2.3	0.300
Effectiveness (/4)	2.3	2.3	0.422
Overall satisfaction (/4)	3.0	3.0	0.139

### Practice activity and costs

Data for 1024 patients were entered in practice databases; 648 before 366 after the workshops. The mean number of GP consultations for back pain before (1.65 [SD 1.342]) and after (1.81 [SD 1.350]) the learning workshops were very similar (p = 0.0784, 2-way unpaired t-test] as was the mean age of patients (Cohort 1: 47.27 SD16.960, range 9-88, Cohort 2: 48.13, SD16.971, range 14-81), p = 0.437 [2-way unpaired t-test]). The activity rates and attendant costs per patient can be seen in Table 5.

**Table 5:** Activity rates_(__μ) _recorded by 9 practices before and after learning workshops with attendant costs_(Ω)_

	Before workshops (n = 648)		After workshops (n = 366)			Before workshops (n = 648)		After workshops (n = 366)	
**Activity**	**Rate**	**Cost/patient (£)**	**Rate**	**Cost/patient (£)**	**Activity**	**Rate**	**Cost/patient (£)**	**Rate**	**Cost/patient (£)**

**In practice**					**Sick certification**				
**Self-Help Literature**	0.116	0.162	0.156	0.218	Given Med 3, 5 or 6	0.263	0.000	0.314	0.000
**Self-help advice**	0.003	0.077	0.000	0.000	**Treatment modalities**				
**Exercise advice**	0.008	0.193	0.003	0.068	Ultrasound	0.002	0.116	0.003	0.205
**Lifesyle advice**	0.002	0.039	0.000	0.000	Epidural	0.026	3.416	0.003	0.355
**Advice about attending physio**	0.005	0.116	0.000	0.000	Other injection	0.003	0.464	0.000	0.000
**Contraceptive leaflet**	0.000	0.000	0.000	0.000	**Consultant referral**				
**Referral for investigations**					Gastroenterology	0.003	0.519	0.003	0.459
**X-ray**	0.053	0.946	0.063	1.131	Gynecology	0.003	0.448	0.003	0.396
**MRI**	0.009	2.921	0.005	1.721	Cardiology	0.005	0.900	0.008	1.590
**Blood test**	0.002	0.031	0.000	0.000	Rheumatology	0.020	5.184	0.027	7.049
**Referral to community services**					Rheumatologist acute back pain service	0.012	3.190	0.000	0.000
**Stop smoking clinic**	0.012	0.495	0.005	0.219	Back Pain Clinic	0.023	4.544	0.036	6.962
**Other GP**	0.005	0.515	0.000	0.000	Hospital Spinal Assessment Clinic	0.002	0.235	0.000	0.000
**Physiotherapy**	0.189	20.176	0.172	18.418	Geriatrics	0.000	0.000	0.003	0.773
**Private Physiotherapy**	0.009	0.371	0.000	0.000	Vascular surgeon	0.000	0.000	0.003	0.473
**Chiropractor**	0.008	0.425	0.011	0.601	Neurologist	0.005	0.946	0.008	1.672
**Private Chiropractor**	0.002	0.085	0.000	0.000	Urologist	0.003	0.495	0.000	0.000
**Osteopath**	0.020	1.005	0.000	0.000	Orthopaedic surgeon	0.040	6.108	0.044	6.645
**Private Osteopath**	0.005	0.232	0.000	0.000	Colorectal surgeon	0.002	0.278	0.000	0.000
**Exercise therapy**	0.005	0.139	0.000	0.000	General surgeon	0.005	0.751	0.011	1.770
**Acupuncture**	0.002	0.085	0.003	0.150	Plastic surgeon	0.002	0.207	0.003	0.366
**Private acupuncture**	0.002	0.085	0.000	0.000	ENT	0.009	1.122	0.000	0.000
**Dietician**	0.002	0.062	0.000	0.000	Opthalmology	0.003	0.328	0.005	0.579
**Midwifery**	0.002	0.077	0.000	0.000	Dermatology	0.006	0.736	0.005	0.650
**Counsellor**	0.003	0.124	0.000	0.000	Psychiatry	0.002	0.377	0.000	0.000
**Contenance nurse**	0.000	0.000	0.003	0.109	**Unspecified**				
**Rehabilitation**	0.000	0.000	0.003	0.109	Further care	0.026	1.445	0.071	3.907
**Community matron**	0.002	0.062	0.000	0.000	**Hospital admission**				
**Pulmonary rehab**	0.002	0.062	0.000	0.000	Emergency admission	0.016	1.597	0.008	0.844
**Other**	0.006	0.247	0.005	0.219	Other admission	0.014	1.391	0.008	0.820

Rate (μ) = number of occurrences/number of patients in cohort. Cost/patient (Ω) = rate x NHS tariff

The main activities recorded as taking place within practices before the workshops were the provision of sickness certification (26.3%) and self-help literature (11.6%). Referrals were spread over 19 different community practitioner groups and 19 consultant groups in secondary care. Changes in rates of referral for X-ray, MRI and hospital admissions, although important, represented only small proportions of patients.

Fifty-three different activities were used, ranging from advice given by doctors, to referrals for investigations or treatments, sickness certifications and hospital admissions recorded. Overall, there was a significant reduction in the number of individual activities used (30/53) after compared to before (48/53) the workshops (p = 0.0001 Fisher exact test). However, the rates for the most prominent activities (rates >0.050) of giving literature, referral to a physiotherapist, chiropractor or osteopath, sickness certification and consultant referral did not change significantly after the workshops (p > 0.05 Fisher Exact test). There were significantly fewer referrals to an osteopath and less use of epidurals after the workshops (p < 0.05 Fisher exact test), but these represented less than 5% of patients and the latter was largely attributable to just one practice.

The highest costs (i.e. over £5 per patient) were for referrals to physiotherapy, orthopaedics and rheumatology, where referral rates (and therefore costs) did not change significantly. The total cost of all activities for a patient episode of care before the learning workshops was £104.78 and £103.70 afterwards.

## Discussion

### Strengths and limitations of the study

A significant strength of this project was the extent to which it engaged both practice staff and patients in the intervention, where the feelings and preferences of both had a significant role in the intervention. Conversely, greater application of the evidence in management; (for example, greater follow-up and reassessment, reassurance, explanation and advice about re-activation) might have been sacrificed to this.

All that was actually done to individual patients could not be recorded by busy GPs and entries in practice databases were not independently validated. Therefore, some evidence-based interventions may have been used, but not recorded. This is a limitation of the study. It is also important to bear in mind that the patient study sampling operated inclusion and exclusion criteria, whereas the practice database study recorded visits by patients with all manner of complaints, of which back pain may have been only one.

### Patient outcomes

The Clinical Value Compass was a helpful tool to frame the outcomes selected, representing a balanced set of outcomes. Although overall functional status and disability were no different after the intervention, as was also found by Cherkin et al, adding inter-professional improvement learning and networking that included patients to learning about evidence-care for back pain had some positive effect on disability scores in female patients when prognostic factors were controlled for [12]. The most influential of these were downheartedness, and leg pain. (Table 2) For males, having a higher baseline RMDQ score had a small, but statistically significant negative effect which, along with downheartedness and self-rated health, was also present for both sexes when the data were pooled. The importance of these prognostic factors has also been noted in other studies [41].

### Practice activity

General practitioners recorded the use of a large number of services to investigate and treat patients with low back pain. Some of these would have been aimed at co-morbidities that accompanied the complaint. It is reasonable to expect that these could not always be disaggregated in the back pain consultation. Additionally, practices were asked to record data for all patients with back pain, including all three triage categories of non-specific back pain, nerve root pain and back pain arising from serious pathology. Thus, and as mentioned above, many patients would not have attended for back pain alone, as reflected in the variety of specialist health personnel they were directed to.

Like previous studies by Engers et al [15] and Cherkin et al [12], we did not find statistically significant differences in the frequency of advice, explanation or information recorded by the general practitioners on practice databases after the intervention. This phenomenon was also reported by McIntosh and Shaw in a qualitative study of patient preferences and expectations of general practitioner care for back pain [42]. GP consultations per patient also did not increase significantly after the workshops, giving little scope for increased active management or follow-up. There was also no significant difference in the main referral destinations after the workshops. The main referral destinations (1/4 of patients) were for some form of physical rehabilitation, but this proportion also did not change after the workshops.

Barnett et al have suggested that guideline recommendations are less likely to be adopted if patient management techniques or referral routes need to be developed [43]. A similar scenario appears to have been in play for depression guidelines where practice-based interventions and referral patterns also did not change [44]. This contrasts sharply with guidelines for the early treatment of asthma, where only changes in prescribing were required and adoption was faster than expected [45]. These issues, combined with problems of patient interaction and lack of a biomedical explanation for the symptoms, have made back pain guideline implementation challenging. This challenge may be augmented further if doctor and patient must confront uncomfortable truths that conflict with patient preferences and societal pressures in order to achieve functional improvements [6]. By contrast, a Canadian randomized controlled trial found that if all patients presenting with back pain were referred to a specialist service, there was a small but significantly greater reduction in disability at six month follow-up [46]. The results of the present study suggest that at least some patients who suffer from downheartedness and/or, bothersome leg pain may need such additional help.

## Conclusion

Previous studies have not found GP educational interventions for back pain to be useful. However, these did not include quality improvement learning and the inclusion of patients with the practice teams. Furthermore, practice activity and referral patterns have not been monitored concurrently and important prognostic factors have not always been adequately controlled for when measuring patient outcomes. Importantly, outcome measures need to embrace a multidimensional perspective which reflects the impact of back pain on the sufferer. For this, the Clinical Value Compass offers a useful tool.

In the present study, when downheartedness and leg pain were controlled for, there was a small, but clinically important and statistically significant reduction in post-intervention disability in female patients. For males, all effects were either small or statistically insignificant except that a higher baseline disability score predicted slightly higher disability at follow-up. This apparent gender effect is unexplained and merits further investigation.

The small, positive effects found in this study occurred without any tangible change in practice activity or patient referral patterns. However, so large is the economic impact of back pain that with general practice being the most frequent provider of care, GP education that combines evidence-based practice with health improvement methodologies and involves the patients themselves, may yet yield worthwhile additional benefits without additional costs.

## Competing interests

The authors declare that they have no competing interests.

## Authors' contributions

All authors participated in the preparation of the manuscript and read and approved the final version.

## Pre-publication history

The pre-publication history for this paper can be accessed here:

http://www.biomedcentral.com/1471-2474/12/28/prepub

## Supplementary Material

Additional File 1 Annexe. Content of LIMBIC Workshops Click here for file
