# Supplementary material for: Back pain outcomes in primary care following a practice improvement intervention:- a prospective cohort study
Source: BMC Musculoskelet Disord. 2011 Jan 27;12:28. doi: 10.1186/1471-2474-12-28 (PMC3040163; doi:10.1186/1471-2474-12-28)
Supplement: Additional File 1 — Annexe. Content of LIMBIC Workshops [file 1471-2474-12-28-S1.DOC]

**Annexe** LIMBIC Workshops – content

| **No.** | **Knowledge base** | **Style** | **Improvement learning** | **Style** |
| --- | --- | --- | --- | --- |
| One | Evidence for the management of back pain | Presentation | The improvement model, process mapping | Presentation |
|  | Role play around the patient consultation | Role play | Developing process maps | Group work |
|  | Group work around successes and challenges | Uniprofessional groups |  |  |
| Two | Doctor and patient expectations | Presentation | Process maps, high level aims | Discussion |
|  | Communication skills | Presentation | Next steps, considering ideas for change | Discussion |
| Three | The encounter and the evidence | Presentation | Progress reports, brief back examination | Uniprofessional groups and plenary |
|  | The encounter and the evidence | Role play demonstration | From ideas to improvement | Presentation |
| Four | GP and patient communication on the initial consultation | Role play using different approaches | Learning about the patient experience | Patient stories |
|  | Wiki demonstration | Practical demonstration | Sharing stories about improvement projects | Team groups paired for discussion then plenary |
|  |  |  | Explanation of use of story boards | Presentation |
| Five | Shift of focus to management of ongoing back pain (6-12 weeks). Presentation of the evidence | Presentation followed by team discussion and plenary | Considerations about next improvement projects and to focus on longer term back pain | Presentation |
|  | Psychosocial issues living with chronic pain | Presentation |  |  |
| Six | Patient biographies to promote discussion about challenge of finding a cure | Patient narratives |  |  |
|  | Supporting patients with continuing back pain | Presentation | Consideration of the application of this new thinking to the next improvement project | Presentation |
|  | Provision of guidelines and evidence from the literature | Handouts |  |  |
|  | Commissioning healthcare and healthcare improvement | Presentation |  |  |
| Seven | Expert Patient Programme | Presentation | Introduction to clinical value compass -balanced set of outcome measures | Presentation and group discussion |
|  | A Back pain service for chronic back pain | Presentation |  |  |
|  | Identification of services available locally for practices | Practice group work |  |  |
| Eight | Presentation of Practice improvement projects | Poster presentations by practice teams | Reflections on learning | Individual and team working |
|  | Psychological perspectives for people with chronic back pain | Presentation |  |  |
